# Impotence from Salicylate of Soda

**Published:** 1881-06

**Authors:** 


### IMPOTENCE FROM SALICYLATE OF SODA.

Dubrisay reports three cases of young, vigorous men, in whom very marked impotence of temporary duration was produced by taking forty-five to fifty-grain doses of salicylate of soda for twenty days while under treatment for rheumatism.—*L'Abeille Med., La Presse Med. Belge.*

